# Early Idiopathic Normal Pressure Hydrocephalus Patients With Neuropsychological Impairment Are Associated With Increased Fractional Anisotropy in the Anterior Thalamic Nucleus

**DOI:** 10.1097/MD.0000000000003636

**Published:** 2016-05-13

**Authors:** Yung-Chieh Chen, Shih-Wei Chiang, Chia-Hsing Chi, Michelle Liou, Duen-Pang Kuo, Hung-Wen Kao, Hsiao-Wen Chung, Hsin I. Ma, Giia-Sheun Peng, Yu-Te Wu, Cheng-Yu Chen

**Affiliations:** From the Department of Biomedical Imaging and Radiological Sciences (Y-CC, Y-TW), National Yang-Ming University; Department of Radiology (S-WC, H-WK, C-YC), Tri-Service General Hospital and National Defense Medical Center; Graduate Institute of Biomedical Electrics and Bioinformatics (S-WC, H-WC), National Taiwan University; Department of Psychiatry (C-HC), Tri-Service General Hospital; Institute of Statistical Science (ML), Academia Sinica, Taipei; Department of Radiology (D-PK), Taoyuan Armed Forces General Hospital, Taoyuan; Department of Neurosurgery (HIM); Department of Neurology (G-SP), Tri-Service General Hospital; Department of Medical Imaging and Imaging Research Center (C-YC), Taipei Medical University Hospital; and Department of Radiology (C-YC), College of Medicine, Taipei Medical University, Taipei, Taiwan.

## Abstract

In this study, we aimed to investigate the reactive changes in diffusion tensor imaging (DTI)-derived diffusion metrics of the anterior thalamic nucleus (AN), a relaying center for the Papez circuit, in early idiopathic normal pressure hydrocephalus (iNPH) patients with memory impairment, as well as its correlation with the patients’ neuropsychological performances. In total, 28 probable iNPH patients with symptom onset within 1 year and 17 control subjects were prospectively recruited between 2010 and 2013 for this institutional review board-approved study. Imaging studies including DTI and a neuropsychological assessment battery were performed in all subjects. Diffusion metrics were measured from the region of the AN using tract-deterministic seeding method by reconstructing the mammillo–thalamo–cingulate connections within the Papez circuit. Differences in diffusion metrics and memory assessment scores between the patient and control group were examined via the Mann–Whitney U test. Spearman correlation analyses were performed to examine associations between diffusion metrics of AN and neuropsychological tests within the patient group. We discovered that early iNPH patients exhibited marked elevations in fractional anisotropy, pure diffusion anisotropy, and axial diffusivity (all *P* < 0.01), as well as lower neuropsychological test scores including verbal and nonverbal memory (all *P* < 0.05) compared with normal control. Spearman rank correlation analyses did not disclose significant correlations between AN diffusion metrics and neuropsychological test scores in the patient group, whereas ranked scatter plots clearly demonstrated a dichotic sample distribution between patient and control samples. In summary, our study highlighted the potential compensatory role of the AN by increasing thalamocortical connectivity within the Papez circuit because memory function declines in early iNPH when early shunt treatment may potentially reverse the memory deficits.

## INTRODUCTION

Idiopathic normal pressure hydrocephalus (iNPH), a well-known debilitating neurological disorder among the elderly population, is characterized by the classic triad of gait disturbance, urinary incontinence, and dementia.^1^ Dementia in iNPH is usually manifested as frontal-subcortical circuit dysfunction.^2–4^ The pathogenesis of memory impairment in iNPH may be due to the tangential shear stress from the intraventricular hyperdynamic cerebrospinal fluid pulsation to the periventricular projection fibers and its associated subcortical structures, thereby disrupting the integrity and functions of important neural circuits that support normal cognitive and memory function, such as the Papez circuit.^3–6^

A central component of the Papez circuit is the anterior thalamic nucleus (AN), which is a specific relay nucleus of the thalamus that plays a pivotal role in modulating emotion and memory function.^7,8^ The AN receives and integrates afferent neuronal inputs from both the hippocampus and mammillary bodies (MBs), principally through the fornix and mammillothalamic tracts (MTTs); moreover, it projects such inputs to the anterior cingulate cortex via the anterior thalamic radiation (ATR). The AN has received considerable attention in recent investigations, particularly in studies regarding memory formation and processing, because of its unique morphological and clinical implications.^9,10^ Here, memory deficit in iNPH may alter the microstructural properties of AN; these alterations are a manifestation of neural plasticity modulated by compensatory recruitment via the Papez circuit in response to declined memory function. Such changes can be quantitatively measured by assessing diffusion metrics derived from diffusion tensor imaging (DTI). DTI is an magnetic resonance imaging (MRI) technique that enables noninvasive measurements of microstructural changes within the brain by characterizing their tensor magnitude, orientation, and anisotropy; thus, DTI offers insights into the microstructural integrity of both gray and white matter.^11–13^ Clinically, diffusion metrics such as fractional anisotropy (FA), mean diffusivity (MD), axial diffusivity (Da), and radial diffusivity (Dr) have been applied in the quantitative analyses of various brain diseases and neuropathological conditions, such as ischemic stroke, brain tumor, neuroinflammation, and neurodegenerative diseases; among these metrics, DTI may play a supportive role in diagnostic assessment, treatment planning, and prognostic evaluation.^14^ In particular, diffusion analysis in iNPH has provided valuable information in terms of differential diagnosis and clinical assessment of symptoms.^15^ Moreover, FA and MD have been used to evaluate cytoarchitectural changes within subcortical regions related to motor function by the pathogenesis of iNPH.^16–19^ In this study, we aimed to assess the changes in diffusion metrics within the AN of early iNPH patients with memory impairment, as well as determine whether such changes are correlated with their neuropsychological performances in terms of memory function.

## MATERIALS AND METHODS

### Study Subjects

This prospective study (conducted between 2008 and 2013) was approved by a local institutional review board (Approval number TSGHIRB-096-05-148-I) with written informed consent obtained from all participants. A total of 28 iNPH patients (13 men and 15 women; mean age ± standard deviation, 75.4 ± 11.1 years; age range, 60–91 years) and 17 healthy control subjects (7 men and 10 women; mean age ± standard deviation, 70.2 ± 6.7 years; age range, 60–81 years) matched for age, sex, and years of education were recruited for this study in Tri-Service General Hospital, Taipei. Years of education (mean years ± standard deviation) were 8.54 ± 4.9 years for iNPH patients and 9.03 ± 5.29 years for control subjects. Patients were diagnosed as probable iNPH within 1 year of onset of symptoms by a clinical neurologist (G.S.P.) on the basis of the diagnostic criteria as per the Japanese guidelines for management of iNPH.^20^ Patient data were excluded from analysis because of (a) incomplete neuropsychological tests; (b) none or incomplete imaging examinations; (c) history of strokes, head trauma, or other neurological diseases; and (d) severe motion-related imaging artifacts. In conclusion, data from 24 patients were included for analysis. For healthy control subjects, the exclusion criteria were as follows: history of neurologic or psychiatric disorders, head trauma or neurosurgery, and history of significant systemic diseases, including cardiovascular disease, diabetes mellitus, and renal function impairment. All subjects underwent neurological testing and clinical evaluation prior to MR imaging and neuropsychological assessments. Detailed demographic and clinical information of participants are summarized in Table 1.

**TABLE 1:**
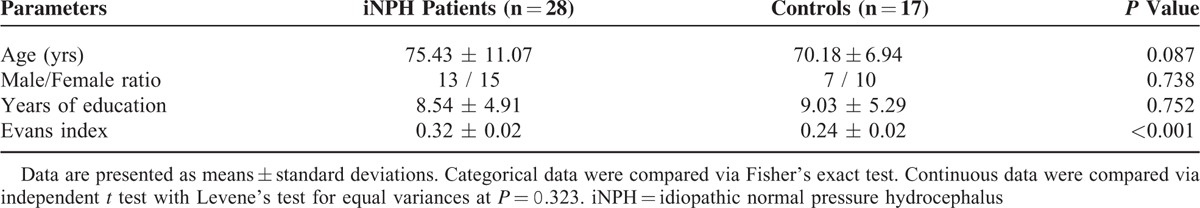
Demographic Features and Clinical Information of Idiopathic Normal Pressure Hydrocephalus Patients and Control

### MRI Study

MRI sessions were performed with a 1.5 T MR unit (GE Signa HDx; General Electric Healthcare, Milwaukee, WI). Conventional sequences included transverse T1-weighted [700/14 (repetition time ms/echo time ms), 1 signal acquired], sagittal T1-weighted (300/14, 1 signal acquired), transverse T2-weighted fast spin-echo (5850/90, 1 signal acquired), and transverse fast fluid-attenuated inversion-recovery (9000/115, inversion time of 2200 ms, 1 signal acquired). The slice thickness for transverse sequences was 5 mm with an intersection gap of 1.5 mm and a 256 × 256 matrix. DTI data were acquired using pulsed gradient spin-echo diffusion-weighted echo-planar sequence with 15 noncollinear diffusion directions plus one B0 image. Sensitivity-encoding parallel imaging with an acceleration factor of 3 was applied to reduce susceptibility distortions. Other parameters were as follows: TR/TE 10,000/85.3 ms, flip angle of 90°, *b* value of 0 and 1000 s/mm^2^, 40 sections, 4 mm-thickness without intersection gaps, field of view of 240 × 240 mm^2^, 128 × 128 matrix zero-filled to 256 × 256, and 2 signals acquired. The resulting DTI voxel size was 0.9375 × 0.9375 × 4.00021 mm^3^. The total acquisition time on each subject was around 15 minutes. All image data were examined by 2 neuroradiologists independently (H.W.K., with 10 years of experience, and C.Y.C., with 26 years of experience) for quality control.

### Neuropsychological Assessment

Standardized neuropsychological tests were performed after each MRI examination session within the same day by a clinical psychologist (C.H.C., 9 years of experience in test design and routine clinical assessment) in the Department of Psychiatry, Tri-Service General Hospital. Neuropsychological measures implemented in this study were focused on evaluating memory function, including both verbal and nonverbal aspects, as well as visuoconstructive ability, naming, and verbal fluency. We evaluated 10 neuropsychological scores. All tests were administered according to the published guidelines. The Chinese version of word list subtest of the Wechsler Memory Scale III (WMS-WL) was used to assess verbal memory in terms of immediate recall, 20 minutes delayed recall, and recognition memory. The Rey complex figure test (RCFT) was used to assess nonverbal memory by means of immediate recall, 20 minutes delayed recall, and recognition memory.^21^ The Wechsler Adult Intelligence Scale III (WAIS-III) was used to assess verbal intelligence quotient (IQ). Visuoconstructive ability, naming, and verbal fluency were assessed with the Chinese version of controlled oral word association test (COWAT). The test results were reported for WMS-WL in terms of standardized scale scores (mean score ± standard deviation, 10 ± 3), RCFT and COWAT using standardized *Z* scores (mean score ± standard deviation, 0 ± 1), and WAIS-III via conventional computation procedures.

### Image Processing and Data Analysis

**FIGURE 1:**
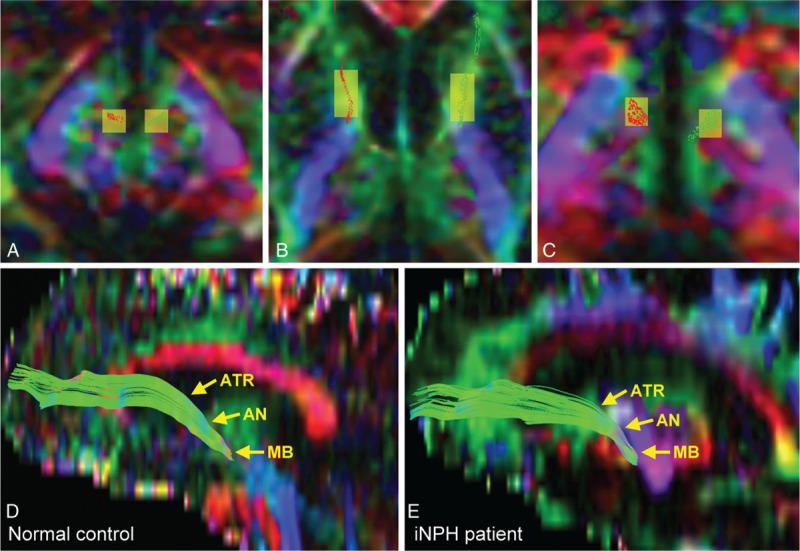
Track-based delineation of the AN in normal control subjects and iNPH patients via DTI, demonstrated on the axial and sagittal planes. In normal control subjects, the 2 deterministic seeds were placed in the mammillary body (A) and the anterior thalamic radiation (B). With this approach, the regions of AN (C) were identified via tracking the mammillo–thalamo–cingulate connections within the Papez circuit (D). The same process was repeated in iNPH patients, yielding highly consistent results on tractography (E). AN = anterior thalamic nucleus.

DTI data were extracted and analyzed (Y.C.C., with 4 years of experience) on an independent workstation. Eddy current correction and skull-striping procedures were conducted with FSL 5.0.6 (FMRIB, Oxford, UK). Diffusion tensor was then calculated to derive whole-brain pixel-wise FA maps. Fiber tracking was performed using MRtrix software package (Brain Research Institute, Melbourne, Australia) on the basis of standard deterministic streamline tractography (500 streamline samples, 0.2 mm step lengths, curvature radius of 2 mm) to identify the region of the AN on each subject's original DTI image space. The level of thalamus was determined by both its fixed location within the diencephalon and relative position with adjacent structures, most notably the internal capsule, basal ganglia, lateral and third ventricles, and the midbrain of the brain stem. Two deterministic seeds (Figure 1), namely, the MB (5 × 5 pixels) and the ATR (6 × 12 pixels), were used to delineate the mammillo–thalamo–cingulate connections within the Papez circuit, consisting of the MTT and the thalamocingulate tract, with the AN situated at the midpoint as a subcortical relay hub. The location and size of the seeds were determined by a priori neuroanatomical knowledge^22^ and then placed in reference to each subject's T1-weighted images. With the proper placement of regions of interest (ROIs) for MB as seed masks and those of ATR as inclusion ROIs, the entire mammillo–thalamo–cingulate projection pathway was produced with high intersubject consistency without the use of exclusion ROIs. The delineation of AN is based on both the tractography results and basic knowledge on human thalamic anatomy. Given that the AN is located at the anterior–superior region of the thalamus, the region of AN (4 × 5 pixels) can be defined by tracking the course of tractography to the corresponding level of the thalamus. After this step, diffusion metrics were then extracted from the region of AN, including FA, pure diffusion anisotropy (*q*), pure diffusion magnitude (*L*), MD, Da, and Dr. *q* and *L* are mathematical indices calculated from eigenvalues, and they constitute the numerator and denominator of FA, respectively. The final output values of each diffusion metric were averaged between both cerebral hemispheres, as laterality in terms of neuropathological involvement and disease severity are generally not expected nor commonly reported among patients with iNPH. A similar analytical approach was adopted by a previous study in iNPH.^23^

### Statistical Analysis

Nonparametric statistical methods were implemented by a clinical statistician (M.L., with 31 years of experience in statistical design). All statistical computations were performed using Statistical Package for the Social Sciences, Version 20.0 (SPSS, Chicago, IL). Between-group differences in diffusion metrics and neuropsychological test scores were examined using Mann–Whitney *U* test. Spearman's rank correlation analyses were performed to evaluate correlations between neuropsychological test scores and diffusion metrics derived from the AN within the patient group only. Statistical significance was set at *P *< 0.05 for all analyses.

## RESULTS:

### Difference in Change in Diffusion Metrics Within the AN of Bilateral Hemispheres Among Subjects

The degree of change in diffusion metrics within the AN of both hemispheres was consistent in both groups of subjects. No significant differences were found by statistical analyses with student *t*-test (for example, the FA values of the AN within the left and right cerebral hemispheres in the patient group were 0.362 ± 0.04 and 0.379 ± 0.059, respectively; *P* = 0.252).

### Between-Group Comparison of Diffusion Metrics Within AN

**FIGURE 2:**
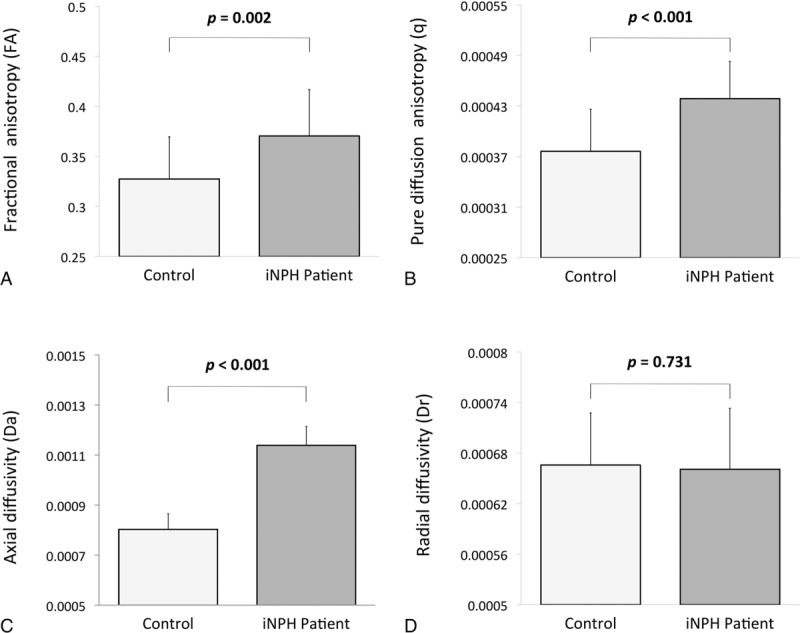
Between-group comparison of diffusion metrics within the AN between normal control and early iNPH patients, including FA (A), *q* value (B), axial diffusivity (C), and radial diffusivity (D). Error bars indicate standard deviation. AN = anterior thalamic nucleus; FA = fractional anisotropy.

Marked differences were found in diffusion metrics of the AN between the patient and control groups (Figure 2). Compared with the control group, iNPH patients presented significantly elevated FA (0.3704 vs. 0.3274; *U* = 88, *P *= 0.002), *q* value (4.39 × 10^−4^ mm^2^/s vs. 3.76 × 10^−4^ mm^2^/s; *U* = 66, *P *< 0.001), and Da (1.14 × 10^−3^ mm^2^/s vs. 8.02 × 10^−4^ mm^2^/s; U = 0, *P *< 0.001). No significant between-group differences were found among the other diffusion metrics, including MD (*P *= 0.459),*L* value (*P *= 0.195), and Dr (*P *= 0.731).

### Between-Group Comparisons of Neuropsychological Test Scores

Comparison analyses demonstrated significant between-group differences in all 10 neuropsychological scores (all *P *< 0.01) (Table 2). Overall, iNPH patients performed markedly worse than controls on all tests, including both verbal and nonverbal memory parameters in immediate recall, delayed recall, and recognition memory.

**TABLE 2:**
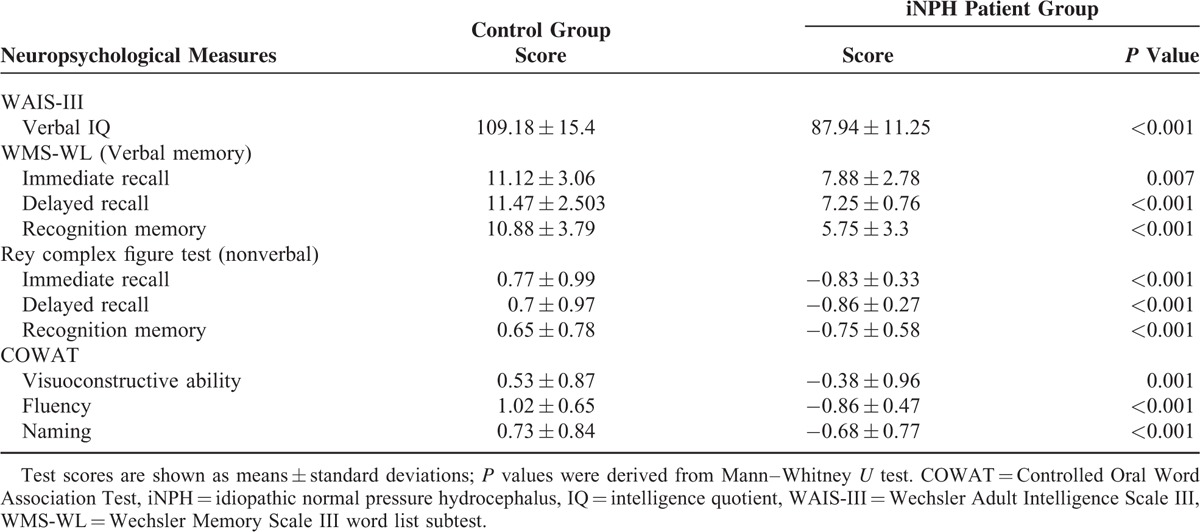
Between-group Comparison of Neuropsychological Test Scores Among Control Subjects and Idiopathic Normal Pressure Hydrocephalus Patients

### Correlations of Neuropsychological Test Scores With DTI Metrics Within the Patient Group

**FIGURE 3:**
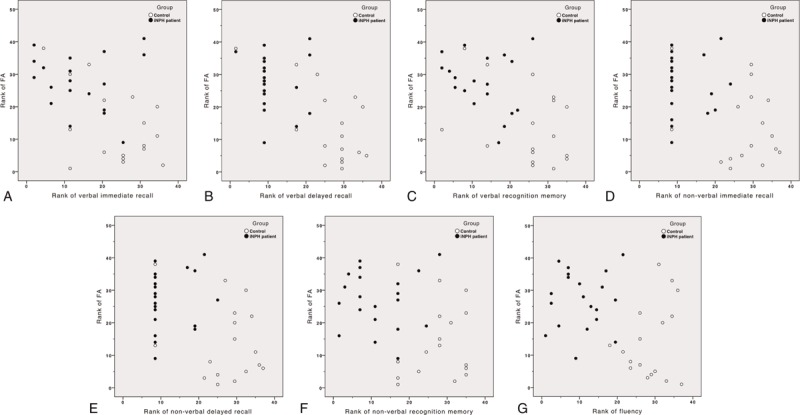
Ranked correlation scatterplot of FA and neuropsychological test scores including data from both patient and control groups. Note the discrete visual separations between the 2 groups of data. White and black dots represent data from control and early iNPH patients, respectively. FA = fractional anisotropy; iNPH = idiopathic normal pressure hydrocephalus.

**FIGURE 4:**
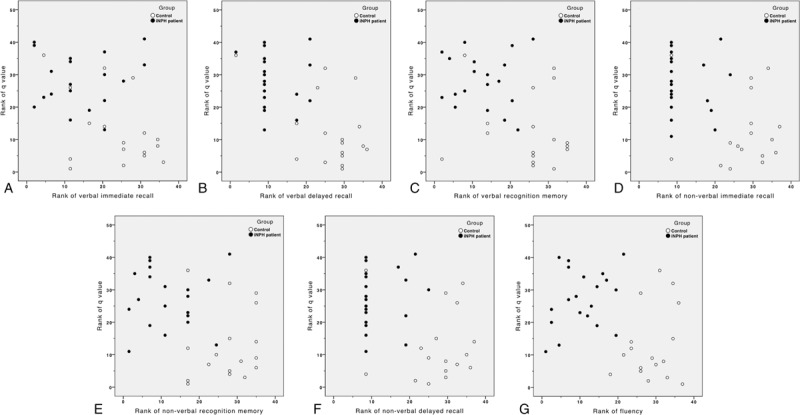
Ranked correlation scatterplot of *q* value and neuropsychological test scores including data from both patient and control groups. Note the discrete visual separations between the 2 groups of data. White and black dots represent data from control and early iNPH patients, respectively. iNPH = idiopathic normal pressure hydrocephalus.

No significant correlations were found between the DTI metrics of the AN and the neuropsychological test scores within the patient group (all *P *> 0.05). However, ranked correlation scatterplot containing data from the patient and control groups revealed dichotic sample distributions (Figures 3 and 4).

## DISCUSSION

In this prospective study, we found significant elevations in selective diffusion tensor metrics, notably FA and *q* value, within the AN of early iNPH patients. As revealed by the neuropsychological tests, the levels of memory performance among iNPH patients were also significantly worse than that of the normal control group. However, the correlation between diffusion metrics of the AN and neuropsychological test scores within the patient group did not reach statistical significance because of the narrow scale of neuropsychological measures and limited sample sizes. Nevertheless, to the best of our knowledge, this study is the first to specifically assess the diffusion properties of the AN, a well-known subcortical structure of significance in memory processing, among patients diagnosed with early iNPH and memory deficits.

The thalamus has been the focus of many neurological and imaging studies in the past, with its individual nuclei now known to be involved in numerous neuropsychological functions. Despite this knowledge, accurately localizing particular thalamic nuclei of interest directly via conventional MRI has been difficult under routine clinical settings; hence, challenges remain to incorporate valuable thalamic information in clinical practice.^24^ Recent improvements in complex image postprocessing algorithms and techniques have enabled automatic delineation and parcellation of major nuclei within the thalamus.^25^ However, such methods are methodologically demanding and limited to subjects without significant neuroanatomical variation or volumetric distortion. Here, we successfully identified the AN within the thalami of both normal and patient group subjects via tract-deterministic nuclei identification, which greatly improves the localization of the AN on individual patient DTI images, while obviating the displacement effects caused by the dilated ventricles. This technique is also an improvement over common methods of placing large ROIs over the thalami, which may inevitably incorporate the diffusivity profiles of multiple thalamic nuclei into analyses, regardless of their significant or nugatory roles in the disease of interest. Incorporation of such profiles into analyses may lead to observations that may not truly reflect the actual disease dynamics within key thalamic nuclei and their influence on various brain dysfunction. In conclusion, the derived anatomic location of AN is also consistent with previous DTI studies.^26^

Compared with the control group, the significantly elevated FA and *q* value within the AN of the iNPH patients were mainly caused by the marked increase in Da, as Dr did not differ significantly between the 2 groups. Increases in Da relative to stable Dr in white matter have been suggested to reflect increases in axon diameter,^27^ whereas increased Da alone was found to reflect the properties of axonal transport and axonal conduction velocity.^28^ The interpretation of diffusional metrics, particularly the FA, Da, and Dr, has been on the forefront of countless DTI studies that argue how the changes in microstructural properties of neuronal structures are reflected by such diffusion markers under various normal and diseased conditions. In axonal conduction, axonal conduction velocity is proportionally determined by axon diameter, which can be evaluated by diffusional metrics such as Da and Dr.^29^ Our findings of increased Da in AN in iNPH patients were consistent with the improved efficiency of conductivity. In light of the dynamic modulatory function of the AN within the Papez circuit, the comparative changes in Da and Dr within the AN among early iNPH patients could reflect increased axonal diffusivity, which corresponds to accelerated axonal conduction velocity and overall conduction efficiency within its thalamocortical projection fibers. On the other hand, FA has been regarded as a measure of microstructural integrity for both white and gray matter, but its significance for the thalamus remains unclear.^30–32^ Previous DTI studies on iNPH attributed the increases in FA within white matter tracts, particularly the corticospinal tract, to the effect of mechanical pressure induced by ventriculomegaly; the changes in FA were also found to be associated with the severity of gait disturbance.^18,19^ Furthermore, compression of frontosubcortical structures has been proposed as the cause of dementia in iNPH.^4^ However, as the thalamus consists of numerous thalamic nuclei with a dense and complex network of projection fibers, it retains a more compact and firm cytoarchitecture.^33^ Therefore, the thalamus is more resilient to deformation under compression, as demonstrated in a study by Osuka et al that thalamic FA does not significantly elevate under mechanical stress.^34^ Other factors that can alter thalamic diffusivity should be considered, because different pathophysiological mechanisms underlying the various symptoms of iNPH may be involved and lead to region-dependent changes in diffusion metrics presentation.^35,36^

Memory impairment in iNPH patients was previously reported in terms of verbal and nonverbal memory recall, fluency, and visuoconstructive ability, with the deficits in recognition memory being less severely affected.^36–40^ Our findings demonstrated that such patterns of functional deficit are present even among early iNPH patients. Therefore, thorough evaluation of patients presented with decline in memory function should be emphasized, along with careful differential diagnoses of iNPH from other common neurological diseases, such as Alzheimer's disease and vascular dementia, as early intervention (i.e., external lumbar drainage and placement of a ventriculoperitoneal shunt) has been demonstrated to significantly improve patient memory performance.^41^ Our patient group also exhibited marked impairments in recognition memory. This finding may correspond the study by Saito and colleagues, who suggested that recognition memory is more involved in iNPH than previously documented,^4^ but further investigations are warranted because our neuropsychological battery design did not incorporate tests that specifically evaluated recognition memory.^42^ Although significant differences in memory performances were found between the 2 groups, correlation analyses between the diffusion metrics within the AN of the patient group and their corresponding neuropsychological test scores did not reach statistical significance. Such incongruities could be partly explained by the limited sample size of our patient group, their individual variations in memory performance, and the neuropsychological tests we adopted to evaluate our subjects. At present, no general consensus has been reached by the international community in the recommendation of specific neuropsychological tests that can best assess the severity of memory deficits among patients with iNPH.^41^ Although several commercially available neuropsychological tests were implemented in our study for the assessment of memory, the lack of wider and finer discrepancies in memory performance scoring, presumably caused by the design of the scoring scales, may have led to the suboptimal ranking of memory performance and potentially hindered the outcome of our study analyses. Such considerations should be taken by future studies aimed at evaluating similar features among patients with iNPH. Nevertheless, the overall distribution of data points from both the patient and control groups, when examined collectively on the ranked correlation scatterplot, allowed for discrete visual separation of patients from the normal controls. This plot feature may assist in the visualization and gross evaluation of the severity of memory impairment among future patients.

The underlying neuroanatomical bases of memory impairment in iNPH are currently unclear. Impairments of the frontal-subcortical projection fibers have been suggested to be involved in the disease process;^36,43,44^ however, the role of the thalamus has not been well explored. The thalamus is known to play a role in reactive upregulation and reorganization of its associated circuits in various diseases. For example, Liu et al found that changes in thalamic diffusivity, notably elevated FA, can reflect increased neuronal activity of thalamocortical pathways in MDMA (or ecstasy) drug abusers.^45^ Tang et al also reported that increases in functional thalamocortical connectivity are correlated with exacerbations of neurocognitive functions in patients with mild traumatic brain injury, which may be associated with thalamic functional reorganization and thalamic network compensatory mechanisms.^46^ From the perspective of the AN, in addition to its capacity to propagate memory-related hippocampal theta rhythms to cortical structures for higher processing,^47^ it modulates neuroplastic properties of its associated projection fibers.^48^ Given the cardinal location of the AN within the Papez circuit and its extensive reciprocal interconnections with other cortical and subcortical structures involved in memory processing, the integrity of the AN is crucial in maintaining synaptic transmission and synaptic plasticity within the anterior thalamic circuitry, as well as supporting the network via dynamic modification of afferent neuronal inputs in reference to preceding activities within the circuit.^49,50^ These notions provide valuable insight into the potential role of the AN in the disease process of iNPH underlying memory impairment. Considering that the integrity of many subcortical structures within the Papez circuit, including the hippocampus,^48^ fornix,^51^ and the ATR,^52^ was previously found to be impaired among patients with iNPH, the AN may undergo reactive microstructural changes at the synaptic level to compensate for the decline in memory performance among iNPH patients and ultimately result in elevated FA and *q* value. This form of neural compensatory mechanism was previously observed in the red nucleus of the midbrain, where increased FA suggests synaptic reorganization during compensatory mechanisms within the corticorubrospinal tract during functional motor recovery among stroke patients; moreover, increased FA is significantly correlated with patients’ motor function score.^31^ Although our current correlation analyses were inconclusive, our preliminary findings may shed light on the potential compensatory role of AN in the disease process of iNPH. Thus, our findings deserve further investigations by future studies.

Several limitations are present in this study and should be considered while interpreting the results. The current technique of delineating the AN within the Papez circuitry on the basis of DTT involves manual operation, which may be prone to possible operator bias. Moreover, this study enrolled a limited number of patients, which could affect the statistical power of our analyses. Therefore, generalization of our results for clinical application may warrant future studies incorporating large patient sample sizes. In addition, this study only evaluated early iNPH patients that had not yet undergone surgical treatment, such as the placement of a ventriculoperitoneal shunt, as well as long-term follow up. Assessment of the evolution of diffusion tensors within the AN following the course of iNPH treatment would be an important extension of the present study in the future.

In summary, this study revealed significant elevations in FA and *q* value within the AN among early iNPH patients, along with marked deteriorations in patients’ neuropsychological performance in memory and verbal fluency. Our results may shed light on the possible compensatory role of the AN in the modulation of memory impairment in iNPH, as well as the prospective diagnostic value of diffusion metrics to assess memory functions in early iNPH patients.
